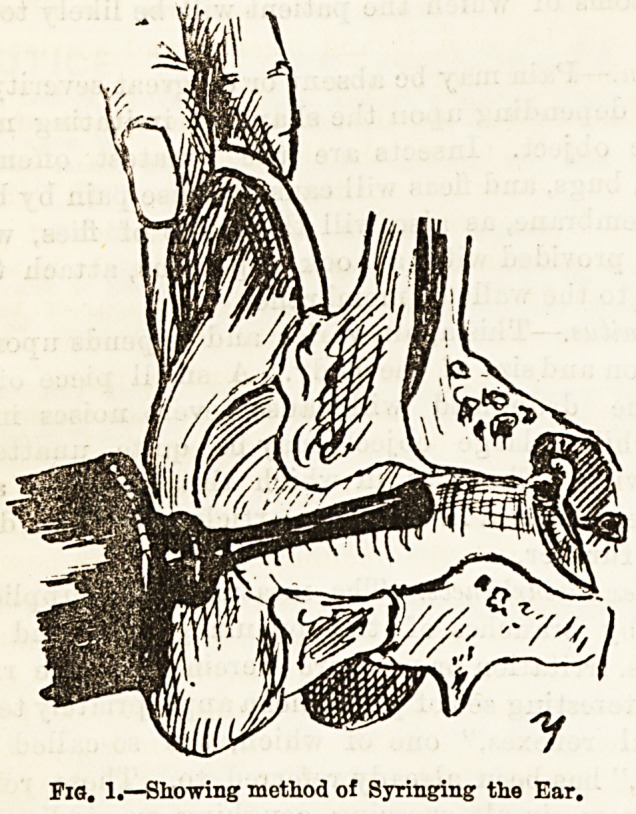# Diseases of the Ear

**Published:** 1894-07-21

**Authors:** P. Macleod Yearsley

**Affiliations:** Aural Surgeon and Surgeon, Farringdon General Dispensary, Assistant Demonstrator of Anatomy and Curator of Museum, and formerly Aural Clinical Assistant to Westminster Hospital


### Medical Progress and Hospital Clinics.

[*The Editor will be glad to receive offers of co-operation and contributions from members of the profession. All letters should be addressed to* The Editor, The Lodge, Porchester Square, London, W.]

### DISEASES OF THE EAR.

By P. Macleod Yearsley, F.R.C.S.Eng., Aural Surgeon and Surgeon, Farringdon General Dispensary, Assistant Demonstrator of Anatomy and Curator of Museum, and formerly Aural Clinical Assistant to Westminster Hospital.

### IV.—Foreign Bodies in the Ear.

The general practitioner is often called upon to remove a foreign body from the ear, a proceeding apparently simple, but frequently presenting considerable difficulty. With care and some anatomical knowledge the proceeding need cause no trouble, yet it is surprising how numerous are the mistakes which arise and the injuries inflicted from carelessness or ignorance. I think that, unless the circumstances be exceptional, no surgeon need fail provided he keeps in mind the anatomy of the meatus, and takes as his golden rule that, unless specially indicated, the syringe should alone be his instrument. As regards the former, there are two spots in the external meatus at which a foreign body is most likely to lodge—at the narrowest part (the isthmus), situated at the inner third of the tube, and in the acute angle formed between the tympanic membrane and the floor of the meatus by the inclination of the former, to which the name *sinus of the meatus* has been applied. Obviously it is the smaller objects only which become lodged in the sinus.

Foreign bodies may enter the ear in various ways, by accident, by design, or, as is the case with insects, by their own efforts; in cases of otorrhœa, flies, attracted by the smell of the pus, have been known to lay their eggs in the meatus. The objects which have been found naturally present great variety, and the best classification is that based upon their behaviour towards the ear. They may thus be divided into three groups:—(1) Bodies which tend to swell; (2) bodies which irritate but do not swell; and (3) bodies which neither irritate nor swell. To the first class belong such objects as peas, beans, bread, wool, wood, sponge, etc., all of which swell by the absorption of moisture; the second group comprises most of the insect tribe, and bodies which irritate either by their chemical properties or large size; the greater number of objects (shot, stones, beads, etc.) belong to the third class.

The symptoms which may arise are as variable as the bodies which cause them. It is not at all uncommon to find cases in which they are conspicuous by their absence. On the other hand, they may be so severe as to considerably alarm the patient. Briefly, the chief manifestations may be put down under four heads: deafness, pain, tinnitus, and reflex disturbances.

*Deafness.*—The amount of deafness of which a foreign body may be the immediate cause is not as a rule great, depending on its size. If it be not removed, the impairment of hearing may become much more marked from inflammatory swelling, the supervention of otitis, or the accumulation of wax round the body as a nucleus. Whenever the deafness is considerable, there will in all probability be other and more distressing symptoms of which the patient will be likely to complain.

*Pain.*—Pain may be absent or of great severity, the latter depending upon the shape or irritating nature of the object. Insects are the greatest offenders; gnats, bugs, and fleas will cause intense pain by biting the membrane, as also will the larvæ of flies, which, being provided with a hook apparatus, attach themselves to the walls or membrane.

*Tinnitus.*—This again varies and depends upon the position and size of the body. A small piece of wax on the drumhead will cause severe noises in the ear, while a large object may be quite unattended therewith. The way in which this symptom arises will be discussed in a future article, and need detain us no further.

*Reflex disturbances.*—The meatus being supplied in part by branches of the pneumogastric and fifth nerves, irritation or pressure therein may give rise to the interesting set of phenomena appropriately termed "aural reflexes," one of which, the so-called "ear cough," has been already referred to. These reflexes vary from simple sneezing, coughing or giddiness, to vomiting, fainting, or even epileptiform convulsions. Their occurrence must not be forgotten, and their recognition may save the practitioner from much vexation and annoyance.

Having given the above very brief outline of the symptoms to which a foreign body may give rise, a few words must be said as to physical examination before proceeding to treatment. The hearing tested by watch and tuning fork, a speculum will be used. Now it is that one is so liable to go wrong, and those who have had any experience will know that no apology is required for dwelling so long on what is an apparently trivial matter. The pinna must be well pulled up, that the meatus may be made as straight as possible, and the sinus brought well into view. Then any difficulty in seeing the object is reduced to a minimum. Although, not strictly foreign bodies, collections of wax may be referred to here, since their treatment is similar and they give rise to much the same symptoms. The appearance of cerumen as seen through a speculum varies in remarkable degree. In children it may be so fluid and foul-smelling as to resemble otorrhœa, in older persons it may be brown and soft, or black and hard. Sometimes it takes the form of a thin white plate, looking like an intact or perforated membrane, while it occasionally presents a curiously honey-combed appearance which may lead the surgeon to think that the membrane has been destroyed, and that it is the inner wall of the tympanum with which he has to deal. Needless to say, a recognition of this diversity of form is of the highest importance in diagnosis and prognosis.

Once recognised, cerumen and foreign bodies call for extraction. Should any inflammatory swelling be present with the latter, it may be necessary to allay it before removing the body, but in most cases it is well to get rid of the cause first, which may be done by gentle syringing. In cases of hard black wax (especially of long standing), the instillation for a day or two of a weak alkaline solution may be required to soften it.

Nearly all foreign bodies can be removed by syring-

Fig. 1.—Showing method of Syringing the Ear.

ing, particularly if it be done when the patient is reclining with the affected ear downwards. Syringing thus from below, gravity contributes to the desired effect. The pinna should be pulled backwards and upwards with one hand, while the nozzle of the instrument is gently introduced and pointed towards the meatal roof, so that the stream of water, washing over the membrane and out along the floor, is not made to strike directly on the sensitive drumhead (fig. 1). The water used should be comfortably warm and carbolised, and during the operation the ear should be examined from time to time to ascertain the progress made. When the meatus is clear it should be carefully dried with a probe armed with wool, and the patient should be directed to wear a small plug of wool for the next twenty-four hours. Neglect of this precaution may result in acute otitis or myringitis.

In a certain number of cases something more than syringing is required. With insects, the offenders may be floated out with warm oil, or first killed by chloroform vapour, tobacco-smoke, or the instillation of some weak germicide that will not harm the ear. The larvæ of flies, when killed, require extraction with forceps, as they cling to the walls of the meatus. When the foreign body belongs to the class of those which swell, syringing may be contra-indicated; in such cases it must be borne in mind that no instrumentation should be resorted to save under proper focal illumination. With a good light, forceps, Lister's hook, or a wire snare may be used with impunity, provided they are manipulated with gentleness. Should much difficulty be experienced, force must never be used; and, unless it be decided to employ an anæsthetic, as is sometimes advisable, it is only by patience and perseverance that we can succeed in the extraction.

## Figures and Tables

**Fig. 1. f1:**